# Compound Comminuted Fracture of Maxillary Bones

**Published:** 1867-06

**Authors:** J. M. Snyder

**Affiliations:** Maple Row, Romney, Hampshire County, Va.


### ARTICLE IX.

### *Compound Comminuted Fracture of Maxillary Bones.*

By J. M. Snyder, M D., Maple Row, Romney, Hampshire County, Va.

On the evening of the 17th of April, Capt. Richard Sloan was kicked by a horse upon the right side of the face, fracturing the lower jaw-bone the foot of the horse came in contact with tbe lower jaw-bone, in a horizontal direction, the full force of the blow being received between the symphysis and right mental foramen. The fracture extended in an oblique direction from the external surface of the jaw, at the first molar tooth internally, to the spina interna , without displacing any of the teeth—except perhaps, slightly, the cuspid tooth. The internal end of the fracture, involving a portion of the spina interna, was comminuted, and a small portion of the bone came away during the progress of the case. There was another fracture between the lower edge of the coronoid process and the first molar tooth ; and in the space of the attachment of the mylohyoides muscle, which was distinctly transverse, and could be easily discovered by the very uneven form of the internal surface of the jaw, and the distinct irregularity of the alveolar arch. This fracture was also comminuted, but no pieces of bone came away. The fracture could be very readily reduced ; but to retain it in coaptation was found to be the great difficulty. The bandages in common use for such cases, and flat corks between the teeth, together with a covering of pasteboard adapted to the shape and made to fit the jaw, were used for a few days, but very unsatisfactorily ; so that it was evident great deformity must ensue unless some more effectual method were soon adopted to prevent motion and insure permanent coaptation. The great severity of the kick produced considerable concussion and the usual concomitant symptoms, though not alarming in their character.

The Captain recollected nothing of the accident, and only remembered having been in the stable with the horse. No abrasion of the facial integuments was discernable until after some days, when a slight contusion was observed opposite the cuspid tooth, with slight ecchymosis.

The superior maxillary bone was also fractured, but not extensively ; three of the teeth, viz., two incisors and cuspid, were forced from the alveolus, and the aveolus itself was crushed to the extent of one and a half inches immediately above the anterior fracture of the lower jaw. The palatine plate was also implicated in the fracture, but without loss of substance.

I found the patient, five or six hours after the accident, suffering intense pain in the fractured part, which was much swollen. I supposed the pain was of a neuralgic character, rather than the result of any inflammatory action, for the circulation had not as yet reacted. This pain, I had no doubt, was kept up by the irritation caused by displacement of the fractured bones, and would subside after the fracture was adjusted, which I found to be the case. About twenty hours after the accident the system reacted powerfully, requiring depletory measures. VS. xxiv. ounces, saline catharthics administered for a few days, together with spts. nit. dulc. and tart. ant. et potassa, and the antiphlogistic treatment generally, until the inflammatory symptoms subsided, when I proceeded to adjust the fracture in a more permanent manner. For this purpose I had made, by a silversmith a thin silver plate, half an inch in width, and sufficiently long to reach from the posterior external part of the second molar tooth on one side to the same point on the other side, covering the symphysis in front, and perforated with very small holes, the width of each tooth apart, and three sixteenths of an inch from the top of the plate. After flexing it so that it fitted precisely the shape of the jaw, I applied it, and with the aid of an assistant succeeded in placing a very small silver wire around the tooth at each extremity of the plate, and drawing each end of the wire through the holes in the plate, and through the small tubes of a double silver canula, twisted them until the bone was drawn to its normal position. Wires were also attached to some of the incisors, for the purpose of keeping the plate more permanently fixed. The plate being elastic, a constant traction was kept up from within outwards, thereby counteracting the displacing action of the mylohyoideus, pterygoid, and masseter muscles. The plate was permitted to remain fifteen or eighteen days, when it was removed. During the progress of the case, to correct fetor and encourage healthy action in the mouth, the solution of chloride of soda was frequently used with a very happy effect.

The Captain suffered from neuralgic pains of the legs, which ultimately subsided, and in thirty days he was discharged cured.

The points of interest in the above case seem to me to be these:—

1. The bone could not be retained in its natural situation by the usual method of bandaging, on account of the strong displacing action of the submaxillary muscles.

2. There being both an oblique and transverse fracture very near each other, and a distinct natural irregularity in the arch of the teeth, rendered it impracticable to retain the fractured ends of the bone in apposition. It therefore became important that some more effectual method of reduction should be adopted, and the above-described plate suggested itself as the best. The fracture never became displaced after its adaptation, and reunion was rapidly and permanently established in about fifteen or eighteen days.—*American Journal of Medical Science.*

---

